# Integrating neuroprotection, antioxidative effects, and precision medicine in glaucoma management with bioactive compounds

**DOI:** 10.1016/j.biopha.2025.118319

**Published:** 2025-07-18

**Authors:** Yan Zhu, Laxmi Moksha, Rebecca Salowe, Vrathasha Vrathasha, Kenneth Pham, Marine-Ayan Ibrahim Aibo, Roy Lee, Mina Halimitabrizi, Isabel Di Rosa, Joan M. O’Brien

**Affiliations:** Center for Genetics of Complex Disease, Department of Ophthalmology, University of Pennsylvania, Philadelphia, PA 19104, USA

**Keywords:** Glaucoma management, Bioactive compounds, Neuroprotection, Antioxidant therapy, Precision medicine

## Abstract

Glaucoma is a group of progressive optic neuropathies characterized by the degeneration of retinal ganglion cells (RGCs) and their axons, leading to irreversible vision loss or blindness if left untreated. Current glaucoma treatments primarily focus on lowering intraocular pressure (IOP), the only proven method to slow disease progression. However, these treatments do not effectively address RGC loss or promote optic nerve regeneration. Emerging research into both natural and synthetic bioactive compounds offers promising new avenues for glaucoma management. This review explores the multifaceted therapeutic potential of bioactive compounds in glaucoma treatment, focusing on their mechanisms of action in IOP reduction and neuroprotection, as well as antioxidant and anti-inflammatory effects. We further review ongoing clinical trials and discuss significant challenges to clinical translation, including the issues of bioavailability, standardization, long-term safety, and regulatory challenges. Furthermore, this paper highlights the potential of integrating precision medicine into bioactive treatments, emphasizing the value of personalized care based on genetic and phenotypic profiles. Finally, the review discusses the role of combination therapies, which leverage the complementary mechanisms of bioactive compounds and conventional treatments. As research progresses, the development of targeted, bioactive-based therapies could transform glaucoma management, offering more comprehensive and effective options for patients with this vision-threatening disease.

## 1. Introduction

Glaucoma comprises a set of progressive optic neuropathies marked by the deterioration of retinal ganglion cells (RGCs) and their axons, leading to structural changes in the optic nerve head and corresponding visual field loss [1]. This disease is the leading cause of irreversible blindness worldwide, affecting an estimated 76 million people in 2020, with projections suggesting this number could rise to 111.8 million by 2040 [2].

There are several subtypes of glaucoma, including angle-closure glaucoma, normal-tension glaucoma, secondary glaucoma, and primary open-angle glaucoma (POAG), which is the most common form of the disease [3,4]. POAG is painless and asymptomatic in early stages, contributing to its reputation as the “silent thief of sight.” Angle-closure glaucoma, while less common, can present acutely with severe pain and rapid vision loss. Given the complexity of glaucoma, understanding its varied pathophysiology is essential to advancing treatment approaches. Here we will focus on POAG specifically because of its high frequency.

The pathophysiology of glaucoma is complex and multifactorial. Elevated intraocular pressure (IOP) remains the only modifiable risk factor, although not all patients with high IOP develop glaucoma, and not all glaucoma patients have elevated IOP [5]. The pressure increase typically results from impaired aqueous humor outflow through the trabecular meshwork. However, other factors contribute to RGC death, including oxidative stress, mitochondrial dysfunction, neuroinflammation, and vascular dysregulation [6]. Risk factors for glaucoma include advanced age, family history of disease, African ancestry, high myopia, and systemic conditions such as diabetes and hypertension [7]. Additionally, corneal thickness, optic nerve head anatomy, and ocular perfusion pressure play roles in glaucoma susceptibility [8]. The multifactorial nature of this disease underscores the need for treatment approaches that extend beyond IOP reduction.

Conventional IOP-lowering therapies, which remain the only proven method to decelerate disease progression [9], encompass several modalities. Topical medications are typically the first-line treatment and include prostaglandin analogs, beta-blockers, alpha-2 agonists, and carbonic anhydrase inhibitors, all of which either decrease aqueous humor production or enhance its outflow [10]. Laser therapies, such as selective laser trabeculoplasty (SLT), aim to improve aqueous outflow through opening the trabecular meshwork [11]. When medications and laser treatments prove insufficient, incisional surgeries such as trabeculectomy or tube shunt implantation are employed to create alternative drainage pathways for aqueous humor. Additionally, minimally invasive glaucoma surgeries (MIGS) have emerged, offering a better safety profile compared to traditional surgeries, albeit often providing more modest IOP reduction [12].

These existing therapies have several limitations. For example, self-administration of topical medications for glaucoma, such as eye drops, can be challenging and often requires lifelong, daily application, sometimes multiple times a day [13]. These factors contribute to compliance issues among patients. The IOP-lowering benefit of laser treatment can diminish over time, necessitating repeat procedures [14, 15]. Traditional glaucoma surgeries, while effective, carry risks of complications such as infection, hypotony, and accelerated cataract formation[16]. Additionally, these therapies offer no benefit to patients with late-stage vision loss. Moreover, current treatments do not directly address RGC loss or promote optic nerve regeneration [17], leaving an unmet need for patients who continue to progress despite well-controlled IOP. This highlights a critical gap in current therapeutic approaches, as patients continue to face vision deterioration over time despite controlled IOP.

Furthermore, current glaucoma therapies are largely uniform and lack personalization, despite growing recognition that glaucoma is a heterogeneous disease influenced by genetic, vascular, and inflammatory factors. Patients may respond differently to the same IOP-lowering agent, and certain subtypes—such as normal-tension glaucoma—may progress even with normal pressure levels [18,19]. Additionally, while lowering IOP is the standard of care, it fails to target upstream drivers of RGC death, including mitochondrial dysfunction, oxidative stress, and neuroinflammation [20]. As a result, many patients experience continued structural and functional decline even under optimal pressure control. These limitations emphasize the need for adjunctive therapies that can directly protect or regenerate neural tissues and be tailored to the individual patient’s disease mechanisms [21].

Emerging research into bioactive compounds, both natural and synthetic, holds promise to bridge this gap [22,23]. Bioactive compounds—such as citicoline, resveratrol, *Ginkgo biloba* extract, and alpha-lipoic acid—have demonstrated potential neuroprotective, antioxidant, and anti-inflammatory effects that could complement current IOP-lowering therapies [24–28]. These bioactive compounds function through various mechanisms, including enhancing mitochondrial function, reducing oxidative stress, and modulating neurotrophic factors, which can contribute to the preservation of RGCs and the optic nerve. Clinical trials investigating these compounds are ongoing, reflecting a growing interest in their potential to provide more comprehensive and multifaceted approaches to glaucoma treatment [17,29]. The integration of bioactive compounds into glaucoma therapy represents a promising strategy to address the limitations of current treatments and improve outcomes for patients.

Given the limitations of current glaucoma therapies—which primarily focus on intraocular pressure reduction but fail to address neurodegeneration and vision loss—there is a growing need to explore complementary therapeutic strategies [5,17]. Bioactive compounds, both natural and synthetic, offer multifaceted benefits such as neuroprotection, antioxidative effects, and anti-inflammatory properties, which can fill this unmet need [22,23,26]. Furthermore, the integration of precision medicine allows for personalization of these therapies based on genetic risk profiles, improving treatment efficacy. This review aims to comprehensively evaluate the therapeutic potential of bioactive compounds in glaucoma management, emphasize their mechanisms of action, explore the role of precision medicine, and discuss challenges and future directions for clinical translation.

**Fig. 1.**
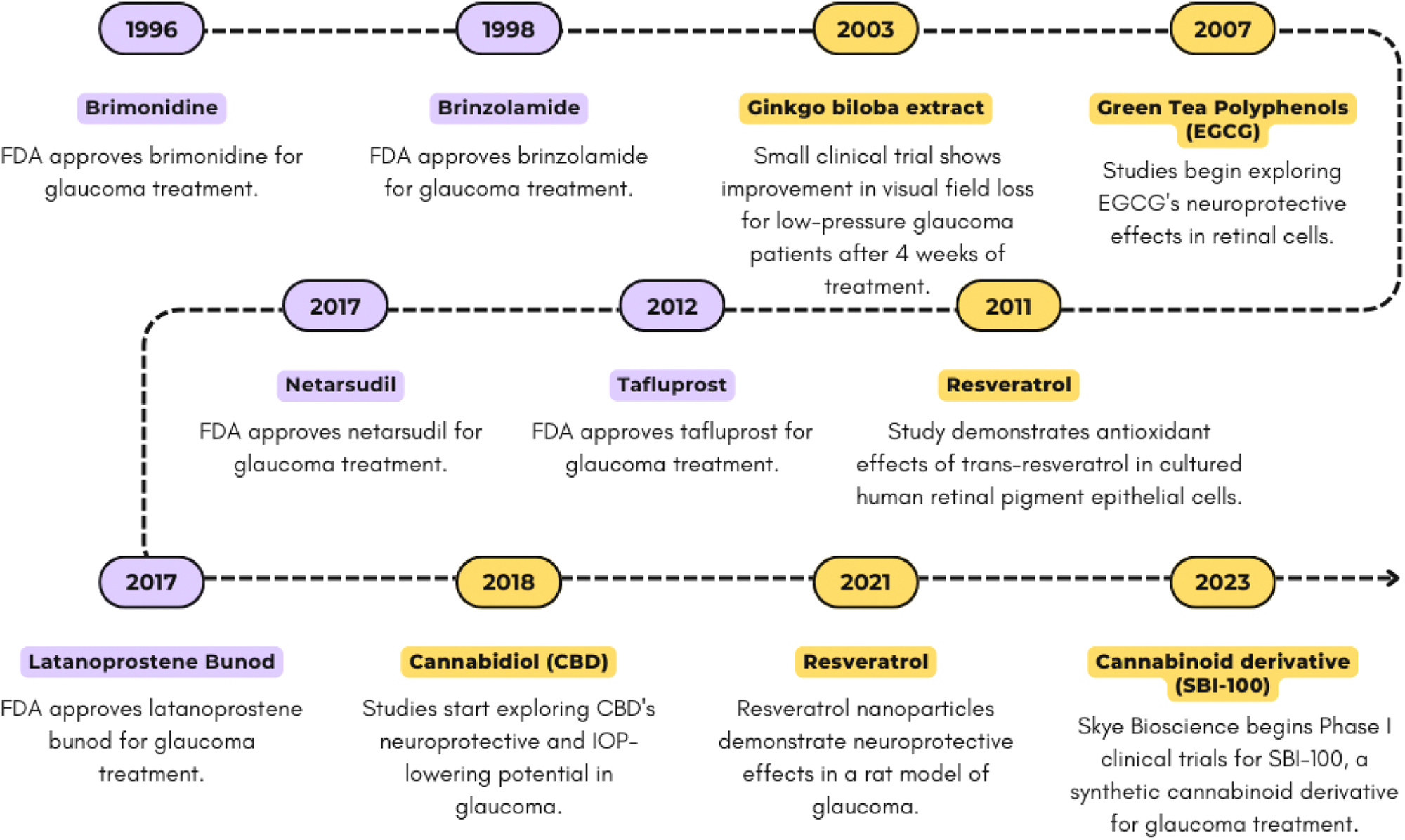
Timeline of Major Bioactive Compounds in Glaucoma Treatment. FDA-approved treatments are shown in purple, while research advances for bioactives are shown in yellow.

Fig. 1 illustrates the key advancements in glaucoma treatment, from the approval of brimonidine in 1996 to the recent introduction of SBI-100 in 2023, marking significant progress in pharmaceutical and bioactive compound development [30–32]. The timeline highlights milestones such as the approvals of brinzolamide and netarsudil, and the exploration of compounds such as *Ginkgo biloba* extract and cannabidiol (CBD) for their neuroprotective and antioxidant properties [23,33,34]. This evolving landscape of glaucoma treatment emphasizes the growing role of bioactive compounds as adjunctive or alternative options to conventional therapies.

This paper will cover both natural and synthetic bioactive compounds, their mechanisms of action, ongoing clinical trials, and the integration of precision medicine approaches. It will also discuss potential challenges in bioavailability, regulatory issues, and combination therapies to provide a comprehensive overview of bioactive-based treatment strategies for glaucoma. By exploring these diverse aspects, this review aims to offer insights into the therapeutic potential of bioactive compounds in glaucoma treatment, particularly focusing on their mechanisms of action for neuroprotection, antioxidant properties, and anti-inflammatory effects. Additionally, it examines current clinical trials and addresses challenges in the clinical translation of these compounds. Ultimately, this review seeks to highlight how bioactive compounds, especially when combined with precision medicine, might lead to more effective, personalized treatments that go beyond IOP control, potentially improving outcomes for patients with glaucoma.

## 2. Natural bioactive compounds in glaucoma

Natural bioactive compounds, with their diverse pharmacological properties, offer significant potential in glaucoma therapy [22,23,35]. They are primarily distinguished by their neuroprotective, antioxidative, and anti-inflammatory activities, which can complement traditional approaches focused on IOP reduction. By targeting various pathological processes such as oxidative stress, neuroinflammation, and neuronal damage, these compounds may provide a multifaceted approach to preserving visual function and retinal health. In the following subsections, we will explore several key natural bioactive compounds, highlighting their mechanisms of action and potential therapeutic roles in glaucoma management. Table 1 summarizes key natural bioactive compounds that have demonstrated therapeutic potential in glaucoma management. These agents, derived from plant, marine, and dietary sources, exert a variety of beneficial effects through antioxidant, anti-inflammatory, neuroprotective, and intraocular pressure-lowering mechanisms. Notable compounds include resveratrol, curcumin, *Ginkgo biloba* extract, and omega-3 fatty acids, all of which have shown promise in protecting RGCs and improving ocular health. The table provides an overview of their sources, mechanisms of action, potential clinical benefits, and reported dosages based on existing literature.

### 2.1. Ginkgo biloba extract

One of the most researched natural compounds in glaucoma therapy is *Ginkgo biloba* extract. *Ginkgo biloba* extract, derived from the leaves of the *Ginkgo biloba* tree, has been utilized in traditional medicine for centuries and holds potential for glaucoma therapy through multiple mechanisms [27]. The extract contains flavonoids and terpenoids with antioxidant properties that may protect RGCs from oxidative stress-induced damage, a critical factor in glaucoma progression [36]. Additionally, *Ginkgo biloba* has vasodilatory effects, potentially enhancing blood flow to the optic nerve [37]. Some studies suggest that *Ginkgo biloba* may help maintain healthy IOP levels, though this effect is less pronounced compared to its neuroprotective properties [38]. Moreover, the extract may help preserve mitochondrial function in retinal cells [39]. Its anti-inflammatory properties could also be beneficial in managing the chronic inflammation associated with glaucoma [40]. While these benefits are promising, ongoing research is required to fully establish the efficacy of *Ginkgo biloba* in glaucoma treatment. Some clinical studies show positive results; for instance, a randomized, crossover trial reported significant improvement in visual field indices in normal-tension glaucoma patients after four weeks of Ginkgo biloba extract administration [27]. However, larger, long-term trials are necessary to confirm these findings and determine the optimal dosage and treatment duration.

### 2.2. Resveratrol

Another promising compound in glaucoma treatment is resveratrol, a polyphenol found in grapes, berries, and peanuts[26]. Resveratrol acts as an antioxidant that can neutralize free radicals in ocular tissues and protect RGCs from oxidative stress-induced damage [41]. Additionally, it reduces inflammation in ocular tissues, mitigating the chronic inflammation associated with glaucoma. This dual antioxidant and anti-inflammatory action make resveratrol a key candidate for neuroprotection in glaucoma.

Resveratrol promotes RGC survival by activating several key signaling pathways: phosphoinositide 3-kinase (PI3K)/Akt Pathway, nuclear factor erythroid 2-related factor 2 (Nrf2)/heme oxygenase-1 (HO-1) Pathway, and sirtuin 1 (SIRT1) Pathway [42]. PI3K/Akt pathway plays a crucial role in cell survival and neuroprotection. Activation of this pathway by resveratrol has been shown to protect RGCs from ischemia-reperfusion injury by reducing oxidative stress and apoptosis. Resveratrol induces the Nrf2 pathway, leading to the upregulation of HO-1. This pathway enhances the antioxidant defense mechanisms in RGCs, thereby mitigating oxidative damage and promoting cell survival. Resveratrol is known to activate SIRT1, a deacetylase involved in cellular stress resistance and survival. Activation of SIRT1 by resveratrol has been associated with neuroprotective effects in RGCs, including the inhibition of apoptosis and the promotion of mitochondrial function.

Resveratrol has shown potential in supporting RGCs through its impact on mitochondrial function and ocular blood flow [43,44]. RGCs, which are highly energy-dependent, require optimal mitochondrial function for survival and resilience, particularly under the stressors associated with glaucoma. Studies indicate that resveratrol enhances mitochondrial health by upregulating proteins critical for mitochondrial stability and function, including sirtuins and optic atrophy 1 (OPA1), thereby bolstering energy production and cellular integrity in RGCs [43]. Furthermore, resveratrol’s vasodilatory properties may positively influence ocular blood flow, an essential factor in retinal health. By increasing nitric oxide production, resveratrol induces vasodilation, which could improve blood flow within ocular tissues, potentially enhancing nutrient and oxygen delivery to the retina [44]. These promising effects make resveratrol an important candidate for further research in ocular blood flow and mitochondrial function in glaucoma management.

Building on its demonstrated biological activity, preclinical studies have explored resveratrol’s therapeutic potential in ocular health, including its ability to lower IOP. Razali et al. investigated the role of adenosine receptors in the IOP-lowering effect of trans-resveratrol in a rat model of steroid-induced ocular hypertension [45]. Their study revealed that topical application of trans-resveratrol at a 0.2 % concentration significantly reduced IOP in normotensive rats, achieving a maximum reduction of 15.1 %. In rats with steroid-induced ocular hypertension, the same concentration achieved a peak IOP reduction of 25.2 %. The study further demonstrated that the oculohypotensive effect of trans-resveratrol is mediated through agonistic activity at the A1 adenosine receptor, as pretreatment with an A1 antagonist abolished this effect. Computational studies supported these findings, showing that trans-resveratrol has the highest binding affinity for the A2B and A1 receptors, followed by A2A and A3, underscoring its potential therapeutic application in glaucoma management. However, despite these promising results, most studies on resveratrol’s effects in glaucoma remain preclinical, conducted in laboratory settings or animal models. Clinical trials in humans are limited, highlighting the need for further research to determine its efficacy, safety, and optimal dosage in a clinical setting.

### 2.3. Curcumin

Curcumin, the active compound in turmeric, has shown potential benefits for glaucoma therapy through various mechanisms [46]. As a potent anti-inflammatory agent, curcumin can reduce chronic inflammation associated with glaucoma. Its strong antioxidative properties enable it to neutralize free radicals, reducing oxidative stress in ocular tissues and protecting RGCs [47]. Curcumin promotes RGCs survival by modulating multiple neuroprotective pathways that enhance cell resilience and inhibit apoptosis. By activating the PI3K/Akt pathway, curcumin fosters cell growth and survival, while simultaneously inhibiting the NF-κB and JAK/STAT pathways, reducing inflammation and apoptotic signaling to protect RGCs in stress conditions like glaucoma [48]. It may help to maintain healthy IOP levels, though this effect is less pronounced than its anti-inflammatory and neuroprotective properties [49]. Additionally, curcumin activates the Nrf2 pathway, leading to the upregulation of antioxidant enzymes such as superoxide dismutase (SOD) and HO-1 [50]. This activation reduces oxidative stress within mitochondria, thereby preserving their function and supporting RGC health. Fibrosis, characterized by excessive extracellular matrix deposition, can impede aqueous humor outflow, leading to increased IOP. Studies have shown that curcumin can inhibit fibroblast proliferation and reduce collagen synthesis, thereby mitigating fibrotic responses [51]. For instance, research indicates that curcumin suppresses the activation of pro-fibrotic pathways, such as transforming growth factor-beta (TGF-β), which plays a significant role in tissue scarring [52].

Curcumin, the active component of turmeric, has demonstrated potential in glaucoma treatment due to its neuroprotective, anti-inflammatory, and antioxidant properties. However, its clinical application has been limited by poor bioavailability, primarily due to low aqueous solubility and rapid metabolism. To address these challenges, recent advancements have focused on enhancing curcumin’s bioavailability through innovative delivery systems. One promising approach involves the development of curcumin-loaded nanoparticles. These nanoparticles improve solubility and protect curcumin from metabolic degradation, thereby increasing its bioavailability. Studies have shown that nanoparticle formulations can enhance curcumin’s therapeutic efficacy in ocular conditions by facilitating targeted delivery to retinal tissues [53]. Additionally, the formulation of curcumin as eye drops has been explored to deliver the compound directly to ocular tissues, bypassing systemic absorption issues. Research indicates that curcumin eye drops can reduce retinal cell loss in experimental models, suggesting potential benefits in glaucoma management [54]. Advances in curcumin delivery systems have significantly addressed the compound’s inherent bioavailability challenges, enhancing its therapeutic potential for glaucoma management. Innovative formulations, such as curcumin-loaded nanoparticles and eye drops, have demonstrated improved solubility, stability, and targeted delivery to ocular tissues, thereby increasing curcumin’s bioavailability and efficacy. While the majority of current research remains at the preclinical stage, these developments lay a robust foundation for future clinical trials aimed at establishing the efficacy and optimal dosing parameters of curcumin in the treatment of glaucoma.

**Fig. 2.**
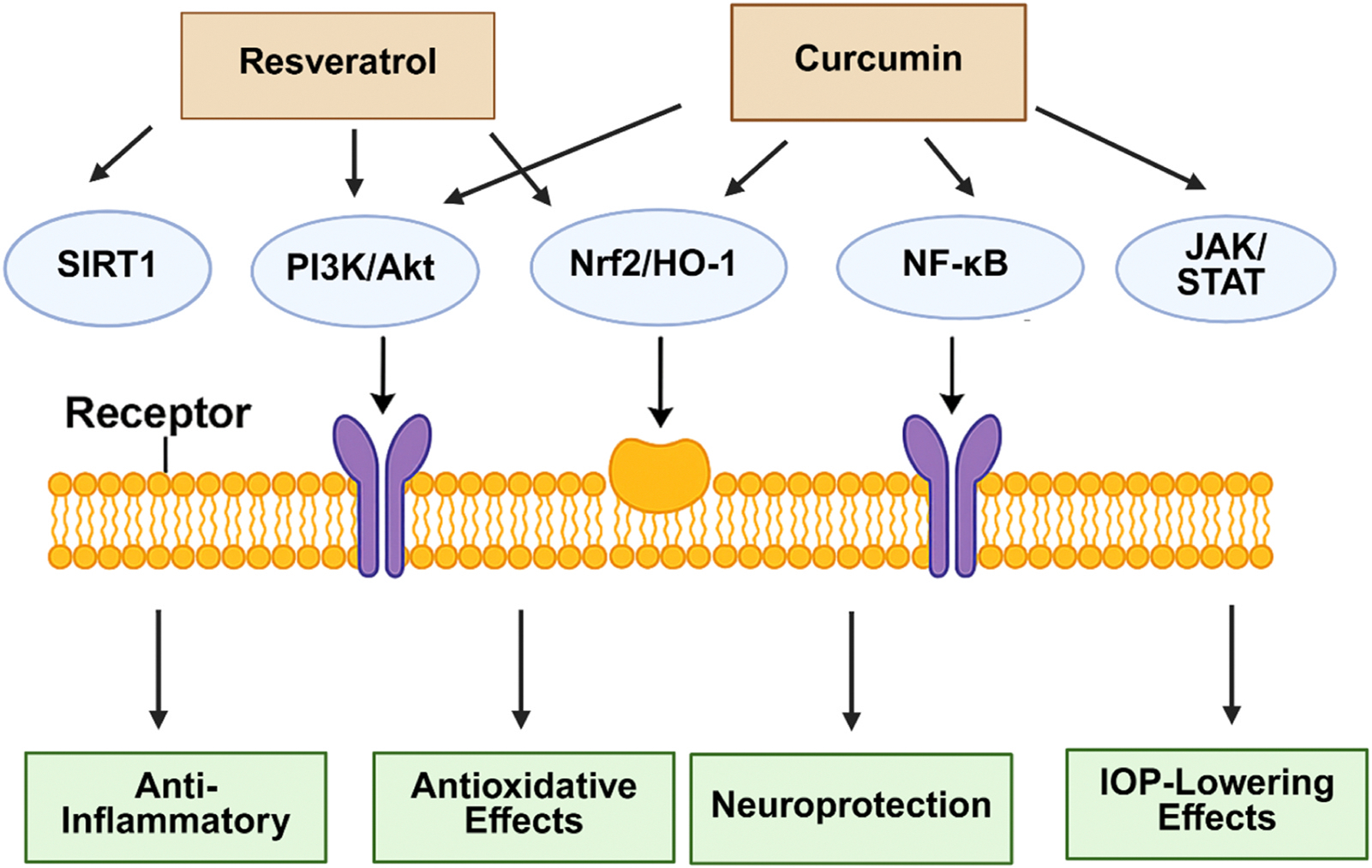
Mechanisms of Action of Curcumin and Resveratrol in Glaucoma Therapy.

Fig. 2 provides a visual summary of the cellular pathways modulated by curcumin and highlights its mechanistic overlap with resveratrol in glaucoma treatment. As illustrated, both compounds activate the PI3K/Akt and Nrf2/HO-1 pathways—key signaling cascades involved in antioxidative stress response and neuroprotection. Curcumin also downregulates pro-inflammatory pathways such as NF-κB and JAK/STAT, contributing to its anti-inflammatory and anti-apoptotic effects. These combined actions culminate in improved RGC survival, reduced oxidative damage, and protection against glaucoma-related neurodegeneration. Fig. 2 integrates these mechanistic insights by showing how curcumin interacts with cell membrane receptors and intracellular mediators to produce its therapeutic outcomes.

### 2.4. Cannabinoids

Cannabinoids, particularly CBD, have been investigated for their potential therapeutic effects in glaucoma due to their ability to reduce IOP and provide neuroprotection to RGCs [55]. Though CBD has a less dramatic IOP reduction effect than tetrahydrocannabinol (THC), it has fewer side effects and still demonstrates neuroprotective properties, slowing RGC damage [55,56]. Its anti-inflammatory properties could help manage glaucoma’s chronic inflammation, and its antioxidant properties reduce oxidative stress in ocular tissues [57].

Research indicates that cannabinoids influence several pathways related to glaucoma: Endocannabinoid System (ECS) [58], WNT/β-Catenin Pathway [59], and Glycogen synthase kinase-3 beta (GSK-3β) Signaling [59]. The ECS, comprising cannabinoid receptors (CB1 and CB2), endogenous ligands, and enzymes, is present in ocular tissues, including the ciliary body, trabecular meshwork, and retina. Activation of CB1 receptors by cannabinoids can reduce IOP by decreasing aqueous humor production and increasing its outflow [58]. CBD has been shown to modulate the WNT/β-catenin pathway, which is involved in cell proliferation and survival. In glaucoma, the downregulation of this pathway is associated with oxidative stress and inflammation. CBD’s activation of the WNT/β-catenin pathway may counteract these detrimental effects, offering neuroprotection to RGCs [59]. GSK-3β is an enzyme that negatively regulates the WNT/β-catenin pathway. Overactivation of GSK-3β has been linked to neurodegenerative processes in glaucoma. CBD inhibits GSK-3β activity, thereby promoting β-catenin accumulation and enhancing cell survival pathways [59].

Despite these promising attributes, challenges remain in integrating CBD into glaucoma treatment protocols. Limited clinical evidence necessitates further research to determine optimal dosages and long-term efficacy. Additionally, the legal status of CBD varies by region, and potential side effects, such as alterations in blood pressure and heart rate, require careful consideration. Healthcare providers should evaluate these factors when considering CBD as a complementary therapy for glaucoma patients [60].

### 2.5. Alpha-lipoic acid (ALA)

Alpha-lipoic acid (ALA), a naturally occurring compound synthesized by mitochondria, serves as a cofactor for enzymes involved in adenosine triphosphate (ATP) production and exhibits antioxidant properties [28]. ALA stimulates the synthesis of glutathione and regenerates other antioxidants, including vitamins C and E, and coenzyme Q10 [61]. As both a water- and fat-soluble antioxidant, ALA can cross cellular membranes, allowing it to distribute to mitochondria where it mitigates oxidative stress and supports cellular energy production [62]. These attributes make ALA particularly well-suited for treating conditions characterized by excessive mitochondrial production of reactive oxygen species (ROS), such as glaucoma [63].

Research has demonstrated that ALA can reduce both small and large blood vessel complications in diabetic models, and it has shown promising results in improving endothelial function and reducing oxidative stress, which may be beneficial in diabetic retinopathy [63]. Additionally, ALA has been shown to enhance the activity of endogenous antioxidant enzymes, such as SOD and glutathione peroxidase, further bolstering the eye’s defense against oxidative damage. By mitigating oxidative stress, ALA helps preserve RGC function and may slow the progression of glaucomatous neuropathy [64]. Furthermore, ALA’s ability to regenerate other antioxidants, including vitamins C and E, amplifies its protective effects against oxidative stress. This multifaceted antioxidant activity makes ALA a promising candidate for adjunctive therapy in glaucoma management [64]. The antioxidant and anti-inflammatory properties of ALA, combined with its ability to regenerate other antioxidants and support mitochondrial function, make it a valuable candidate for glaucoma management [65]. ALA activates the PI3K/Akt pathway, a critical component of insulin signaling, thereby improving glucose uptake and reducing oxidative stress [66]. Additionally, ALA’s metal-chelating properties enable it to bind transition metals like iron and copper, which catalyze the formation of ROS. By sequestering these metals, ALA reduces ROS production and mitigates oxidative damage [67]. These dual actions underscore ALA’s potential as a therapeutic agent in conditions characterized by oxidative stress. Overall, ALA represents a promising bioactive compound for glaucoma therapy, offering potential benefits in neuroprotection and the mitigation of oxidative stress, which are critical aspects of disease management [63,65].

### 2.6. Other natural bioactive compounds

In recent years, attention has been drawn to several other popular natural bioactive compounds, which have shown potential in complementing traditional glaucoma therapies [23]. Bilberry extract, rich in anthocyanins, demonstrates robust antioxidant properties, potentially safeguarding RGCs from oxidative stress and possibly aiding in IOP management and enhancing ocular blood flow [68,69]. Astaxanthin, derived from microalgae, shows potent antioxidant and anti-inflammatory effects, providing neuroprotective benefits crucial for maintaining RGC integrity [70,71]. Green tea polyphenols (EGCG) are noted for their strong antioxidant and anti-inflammatory actions, contributing to reduced IOP and enhanced protection of RGCs and ocular blood circulation [72].

Quercetin, a flavonoid found in onions and apples, provides significant antioxidant, anti-inflammatory, and neuroprotective effects, helping to protect RGCs from oxidative damage and apoptosis while potentially reducing IOP and inflammation [73,74]. Ginseng contains ginsenosides that also exhibit these effects, which could lead to enhanced retinal function [75–77]. Saffron, rich in crocin, is known to modulate inflammatory pathways, protect against RGC apoptosis, and possibly improve retinal health and function [78,79]. Lastly, omega-3 fatty acids have been shown to reduce IOP, protect RGCs, and enhance retinal function through their anti-inflammatory and neuroprotective effects [80,81].

Integrating these natural bioactive compounds into glaucoma management offers a multifaceted approach that extends beyond IOP reduction. By leveraging their diverse mechanisms—such as enhancing mitochondrial function, mitigating oxidative stress, and modulating neurotrophic factors—these compounds provide neuroprotective and regenerative benefits. Notably, many of these substances, including bilberry extract, astaxanthin, green tea polyphenols, quercetin, ginseng, saffron, and omega-3 fatty acids, are commonly consumed in various cultures and have established safety profiles. This familiarity underscores their potential for safe integration into therapeutic regimens. However, to fully harness their benefits in glaucoma care, rigorous research and well-designed clinical trials are essential to confirm their efficacy and safety, ensuring that these promising compounds can be effectively incorporated into standard treatment protocols.

### 2.7. Limitations of natural compounds

While natural bioactive compounds offer promising antioxidant, neuroprotective, and anti-inflammatory benefits, several limitations hinder their clinical translation. First, many compounds such as curcumin and resveratrol suffer from poor oral bioavailability, owing to low aqueous solubility and rapid metabolism [82,83]. Second, standardization issues—including variability in extraction methods, active ingredient concentration, and batch-to-batch consistency—complicate dosage reproducibility [84]. Third, limited clinical trial data are available for many compounds, with most evidence derived from preclinical models. Fourth, potential interactions with other drugs or supplements, as well as the need for long-term safety data, remain underexplored [85]. Overcoming these limitations will require improved delivery systems, robust quality control protocols, and well-designed clinical trials to ensure consistent efficacy and safety in patients with glaucoma.

## 3. Synthetic bioactive compounds for glaucoma treatment

The development of synthetic bioactive compounds for glaucoma treatment represents a significant area of research, aimed at addressing the multifaceted pathophysiology of the disease and overcoming limitations of current therapies. This section focuses on two key categories of synthetic bioactive compounds: 1) novel neuroprotective agents and 2) antioxidants/anti-inflammatory compounds. By exploring these compounds, researchers aim to develop treatments that extend beyond IOP reduction to address the neurodegenerative aspects of glaucoma. Table 2 outlines key synthetic bioactive compounds that are currently under investigation or in clinical use for glaucoma treatment. These agents target diverse mechanisms including neuroprotection, IOP regulation, antioxidant defense, and modulation of neuroinflammation. Compounds such as brimonidine, netarsudil, citicoline, and memantine exemplify a shift toward therapies that go beyond IOP-lowering to protect RGCs and preserve visual function. The table presents each compound’s class, mechanism of action, reported dosage, and potential benefits, offering a comprehensive overview of their therapeutic relevance in glaucoma care.

### 3.1. Novel neuroprotective agents

Several synthetic agents show promise in safeguarding RGCs. Neuroprotection in glaucoma aims to preserve RGCs and their axons, independent of IOP reduction [86]. Several classes of synthetic neuroprotective agents have shown promise in preclinical and early clinical studies [87]. N-methyl-D-aspartate (NMDA) receptor antagonists, such as memantine, have been extensively studied due to their ability to block excessive calcium influx through NMDA receptors, potentially preventing excitotoxicity-induced RGC death [88]. Although a large Phase III clinical trial (Allergan’s memantine trial) did not demonstrate significant benefits in slowing glaucoma progression, the outcome may have been influenced by limitations in study design, patient selection, and endpoint sensitivity. Notably, post-hoc analyses suggested potential efficacy in specific patient subgroups, indicating that further research with refined parameters could uncover meaningful neuroprotective effects [89].

Another well-known compound in glaucoma treatment, brimonidine, demonstrates dual benefits in both IOP reduction and neuroprotection. While alpha-2 adrenergic receptor agonists like brimonidine are well-established for reducing IOP, recent research highlights their potential neuroprotective properties as an added benefit in glaucoma treatment [90]. Brimonidine has been shown to upregulate neurotrophic factors, reduce oxidative stress, and modulate NMDA receptor function [90]. The Low-Pressure Glaucoma Treatment Study indicated that brimonidine was more effective than timolol in preserving visual field function, suggesting a potential neuroprotective effect beyond IOP reduction [91].

Rho kinase (ROCK) inhibitors, a newer class of drugs in glaucoma management, offer potential beyond lowering IOP. ROCK inhibitors, such as netarsudil, have emerged as promising agents in glaucoma management, primarily due to their efficacy in reducing IOP [33]. Clinical studies have demonstrated that netarsudil effectively lowers IOP by increasing trabecular outflow and decreasing episcleral venous pressure [33,92]. In the MERCURY-1 and MERCURY-2 clinical trials, a fixed-dose combination of netarsudil and latanoprost was evaluated for its efficacy and safety in patients with POAG or ocular hypertension (OHT) [92]. The pooled data from these trials indicated that the combination therapy achieved a statistically superior IOP reduction compared to the individual components at all time points assessed over three months. Notably, nearly twice as many patients receiving the fixed-dose combination achieved at least a 30 % reduction from baseline in IOP, as recommended by the American Academy of Ophthalmology for first-line treatment, compared to those on monotherapy [92].

Beyond IOP reduction, ROCK inhibitors have shown potential neuroprotective effects. Preclinical studies suggest that ROCK inhibition may promote RGC survival, axon regeneration, and improve ocular blood flow. These findings are particularly significant, as they address the neurodegenerative aspect of glaucoma, which current IOP-lowering therapies do not fully target [93]. While the neuroprotective benefits of ROCK inhibitors in humans require further investigation, their dual action in lowering IOP and potentially preserving RGCs positions them as a promising advances in glaucoma therapy. Ongoing research and clinical trials are essential to fully elucidate their therapeutic potential and to optimize treatment protocols for patients with glaucoma.

Citicoline is another compound garnering attention for its neuroprotective effects in glaucoma. Citicoline, an endogenous compound involved in phospholipid synthesis, has received attention for its neuroprotective properties in glaucoma management [25]. It is reported to enhance mitochondrial function, reduce oxidative stress, and support cell membrane integrity [24]. Clinical studies have demonstrated that citicoline can improve visual function in glaucoma patients. For instance, a randomized, double-masked, placebo-controlled, cross-over study found that oral citicoline administration led to significant improvements in the composite score of the Visual Function Questionnaire-25 (VFQ-25), indicating enhanced quality of life related to visual function [94]. Additionally, the neuroprotective action of citicoline in glaucoma involves several key mechanisms [95]. Citicoline supports mitochondrial health by stabilizing mitochondrial membranes and improving energy production. This is particularly important for RGCs, which have high energy demands and are vulnerable to mitochondrial dysfunction. By promoting the synthesis of glutathione and other antioxidants, citicoline helps mitigate oxidative stress within ocular tissues. This reduction in oxidative damage is vital for preserving RGCs and preventing apoptosis. In addition, citicoline increases the levels of neurotransmitters such as dopamine and acetylcholine in the central nervous system. This modulation enhances neural communication and may improve visual function in glaucoma patients. Finally, citicoline facilitates the repair of damaged neuronal membranes and supports neuroplasticity, aiding in the recovery and maintenance of neural pathways affected by glaucomatous damage. While these findings are promising, larger, well-controlled studies are necessary to confirm citicoline’s efficacy and establish its role in glaucoma therapy. The broad therapeutic applications of citicoline across various neurodegenerative and ophthalmologic conditions underscore its potential [96], yet systematic reviews highlight the necessity for extensive research to substantiate its benefits specifically in glaucoma treatment [97].

Neurotrophic factor mimetics represent a cutting-edge approach in glaucoma neuroprotection. Neurotrophic factor mimetics are synthetic compounds engineered to replicate or enhance the effects of endogenous neurotrophic factors, such as brain-derived neurotrophic factor (BDNF) and ciliary neurotrophic factor (CNTF), in the treatment of glaucoma [98]. BDNF supports RGC survival and function by activating the TrkB receptor, leading to downstream signaling that promotes cell survival and synaptic plasticity [99]. Synthetic BDNF mimetics aim to activate TrkB receptors, thereby mimicking BDNF’s neuroprotective effects. For instance, small molecule BDNF mimetics have been shown to activate TrkB signaling and prevent neuronal degeneration in preclinical models [99]. Similarly, CNTF promotes neuronal survival through the activation of the CNTF receptor complex, initiating pathways that prevent apoptosis [86]. Encapsulated cell therapy (ECT) devices releasing CNTF have been developed to provide sustained delivery, showing potential in protecting RGCs in glaucoma models. Clinical investigations into neurotrophic factor therapies for glaucoma have highlighted the potential of the NT-501 implant and BDNF gene therapy. The NT-501 implant, an ECT device, delivers CNTF directly to the retina, providing sustained neuroprotective effects. Phase I clinical trials of NT-501 in glaucoma patients have demonstrated that the device is well-tolerated, with exploratory analyses indicating potential benefits in preserving visual function [100]. This implant-based approach addresses the challenge of sustained neurotrophic delivery, offering a continuous source of CNTF to support RGC health over time. Meanwhile, BDNF gene therapy presents another promising avenue, aiming to enhance RGC resilience by promoting the expression of BDNF directly within ocular tissues. Preclinical studies have shown that BDNF gene delivery protects RGCs from degeneration, with observed improvements in cell survival and functionality [101]. Together, these innovative approaches underscore the potential of neurotrophic therapies in glaucoma, offering new avenues for protecting vision by addressing the neurodegenerative component of the disease. Further clinical trials are essential to confirm these findings and to optimize delivery methods, establishing neurotrophic factor therapies as viable tools in glaucoma management.

### 3.2. Antioxidants and anti-inflammatory compounds

Addressing oxidative stress and inflammation is crucial in managing glaucomatous damage, and synthetic compounds are being developed with these specific targets in mind. Oxidative stress and chronic inflammation play significant roles in glaucomatous damage [102]. Synthetic antioxidants and anti-inflammatory compounds are being developed to address these aspects of glaucoma pathophysiology [103]. Novel synthetic antioxidants aim to provide more potent and targeted protection against oxidative stress in ocular tissues. Mitochondria-targeted antioxidants, such as MitoQ and SkQ1, specifically accumulate in mitochondria, which is the primary source of cellular ROS, potentially providing more efficient protection against oxidative damage [104]. Preclinical studies have shown promising results in protecting RGCs from oxidative stress-induced death, but clinical trials are needed to establish efficacy in glaucoma patients [104].

In addition to antioxidants, Nrf2 activators are being investigated as promising agents for enhancing cellular defense mechanisms against oxidative damage. Nrf2 activators, including compounds like bardoxolone methyl and RTA 408, are recognized for their ability to enhance cellular antioxidant defenses by activating the Nrf2 pathway [105]. This pathway plays a pivotal role in regulating the expression of antioxidant proteins that protect against oxidative damage. While these activators have been primarily investigated for conditions such as chronic kidney disease and cancer, their potential application in glaucoma is gaining attention due to the role of oxidative stress in RGC damage [105,106].

For managing inflammation in glaucoma, targeted anti-inflammatory treatments are being increasingly explored to overcome the limitations of traditional therapies. In glaucoma management, traditional anti-inflammatory treatments, such as corticosteroids and nonsteroidal anti-inflammatory drugs (NSAIDs), are commonly employed to mitigate ocular inflammation [107]. While these agents effectively reduce inflammation, their broad-spectrum activity can lead to significant side effects, including elevated IOP, cataract formation, and systemic complications [108]. Moreover, these treatments may not adequately address the specific inflammatory pathways implicated in glaucoma pathogenesis [108]. Targeting TNF-α offers a more precise therapeutic approach [109]. TNF-α is a pro-inflammatory cytokine that plays a pivotal role in neuroinflammatory processes associated with RGC apoptosis in glaucoma [109,110]. Elevated levels of TNF-α have been detected in the glaucomatous optic nerve head and retina, correlating with disease progression [110]. By specifically inhibiting TNF-α, therapies like XPro1595 aim to disrupt this pathogenic pathway, potentially reducing RGC loss more effectively and more precisely than broad-spectrum anti-inflammatory agents[111]. Preclinical studies have demonstrated that TNF-α inhibitors can attenuate RGC death and preserve visual function in animal models of glaucoma [110]. These findings suggest that TNF-α-targeted therapies may offer neuroprotective benefits beyond the capabilities of conventional anti-inflammatory treatments. Additionally, interleukin-1 receptor antagonists, such as anakinra, a recombinant IL-1 receptor antagonist, are also being explored for glaucoma management due to the involvement of IL-1 signaling in neuroinflammation associated with this disease [112].

Recent advancements have introduced a variety of synthetic bioactives targeting multiple pathways in glaucoma treatment. Brinzolamide, a carbonic anhydrase inhibitor, effectively lowers IOP by reducing aqueous humor production, making it a valuable addition to glaucoma management regimens [113,114]. Latanoprostene bound employs a novel dual mechanism that enhances uveoscleral and trabecular outflow, significantly reducing IOP with a favorable safety profile [115, 116]. Sunitinib offers a unique approach by reducing glaucomatous damage through the inhibition of pathological tyrosine kinase activity [117]. Dorzolamide, another carbonic anhydrase inhibitor, similarly lowers IOP by decreasing aqueous humor production, and its long-term efficacy and safety profile make it a mainstay in glaucoma therapy [118–120]. Anecortave acetate, an angiostatic corticosteroid analog, uniquely targets angiogenesis and fibroblast activity, offering another pathway to reduce IOP without the well-known steroid-induced side effects typical of corticosteroids [121]. Tafluprost, a prostaglandin analog, is notable for its ability to increase uveoscleral outflow, effectively lowering IOP with fewer side effects, making it suitable for patients intolerant of other treatments [122,123]. Lastly, ILUVIEN^®^ (fluocinolone acetonide intravitreal implant) represents a significant innovation in sustained drug delivery systems, providing a long-duration control of inflammation and IOP, which is crucial for patients requiring consistent management of their condition [124]. These agents collectively illustrate the progress in targeting multiple aspects of glaucoma pathology through synthetic bioactives.

Despite these promising developments, synthetic bioactives in glaucoma therapy still face several critical challenges. Developing formulations to ensure that adequate concentrations of these compounds reach the retina, and optic nerve remains a significant challenge. The chronic nature of glaucoma necessitates careful evaluation of the long-term efficacy and safety profiles of these novel compounds [125]. Investigating potential synergies between different classes of synthetic bioactive compounds and their integration with existing glaucoma therapies is crucial for optimizing treatment outcomes. Identifying biomarkers that predict response to specific neuroprotective, antioxidant, and/or anti-inflammatory therapies could enable more targeted treatment strategies. Developing appropriate endpoints and study designs to effectively evaluate neuroprotection and disease modification in glaucoma remains challenging but should nevertheless be the goal in clinical trials.

In summary, future research must address these difficulties and expand on the promise of bioactive compounds in glaucoma management. Future research should also focus on developing novel targets and compounds. The development of multi-modal therapies that combine IOP-lowering, neuroprotection, and anti-inflammatory effects may offer the most comprehensive approach to glaucoma management. Synthetic bioactive compounds represent a promising frontier in glaucoma research, offering the potential to address key aspects of disease pathophysiology beyond IOP reduction. As our understanding of glaucoma mechanisms and genetics continues to evolve, the development of targeted synthetic compounds may lead to more effective and precise treatment strategies, ultimately improving outcomes for patients with this sight-threatening disease.

## 4. Precision medicine in bioactive treatment for glaucoma

Precision medicine, a tailored approach to patient care, aims to customize treatments based on individual genetic, environmental, and lifestyle factors [129]. This approach holds significant promise in the management of glaucoma, a complex and multifactorial disease [18, 130]. Integrating precision medicine with bioactive treatments may enhance the efficacy of therapeutic interventions. This section explores the potential of precision medicine in the context of bioactive compounds for glaucoma treatment, examining genetic markers and personalized treatment regimens. By integrating these personalized approaches, clinicians can potentially achieve better-targeted and more effective glaucoma management strategies.

### 4.1. Genetic markers and risk stratification

Identifying genetic markers has become a cornerstone in advancing precision medicine for glaucoma. In recent studies, genetic factors have been shown to play a crucial role in the susceptibility and progression of glaucoma, with notable variations in genetic risk among different ancestry groups [131,132]. Identifying genetic markers associated with the disease can facilitate early diagnosis and targeted interventions [133,134]. Genome-wide association studies (GWAS) have identified numerous loci linked to glaucoma, including the *MYOC*, *OPTN*, and *TBK1* genes [131]. Research indicates that individuals of African descent are disproportionately affected by POAG, with a prevalence approximately five times higher than that in European ancestry populations [135]. In the recent GWAS study led by our team, three novel genetic variants associated with POAG were identified specifically in African ancestry individuals, underscoring the unique genetic architecture of glaucoma across populations [136]. Furthermore, a polygenic risk score (PRS) developed from African ancestry data outperformed a PRS from European ancestry data in predicting POAG risk within African American cohorts, demonstrating the importance of ancestry-specific approaches in glaucoma genetics [136].

Using these genetic markers, clinicians can further refine patient stratification to optimize treatment outcomes. These genetic markers can help stratify patients based on their risk profiles, enabling more precise treatment approaches. For example, individuals with different *APOE* gene variants respond uniquely to neuroprotective treatments [137,138]. APOE ε4 carriers may show reduced treatment efficacy due to impaired amyloid-β clearance [138], whereas APOE ε2 carriers often benefit more from neuroprotective therapies, possibly due to enhanced mitochondrial function and cell survival mechanisms [137]. These genotype-specific responses highlight the importance of personalized approaches in neuroprotection [128]. By understanding these genetic differences, clinicians can select bioactive compounds that are more likely to be effective for each patient, reducing the trial-and-error process often associated with glaucoma management. For example, variations in the prostaglandin F receptor (*PTGFR*) gene have been associated with differing responses to latanoprost, a commonly prescribed medication for lowering IOP in the treatment of glaucoma [139]. Certain single-nucleotide polymorphisms (SNPs) in *PTGFR* can lead to reduced efficacy of latanoprost in patients with POAG [140].

In summary, genetic markers serve as a dual-purpose tool in glaucoma management: they facilitate early identification of high-risk individuals through risk stratification and enable personalized treatment decisions based on predicted drug response or disease subtype. By incorporating genetic insights into clinical workflows, clinicians can tailor bioactive therapies and conventional treatments to an individual’s genetic profile, improving therapeutic efficacy while minimizing side effects. As more ancestry-specific markers are validated, particularly in underrepresented populations, the application of precision medicine in glaucoma will become increasingly equitable and impactful.

### 4.2. Personalized treatment regimens

Personalized treatment regimens are essential to achieving the full potential of precision medicine in glaucoma therapy. The integration of precision medicine into bioactive treatment for glaucoma involves developing personalized treatment regimens based on a patient’s unique genetic makeup and disease characteristics [130,141]. This tailored approach can optimize therapeutic outcomes by considering factors such as drug metabolism, efficacy, and potential side effects. For instance, personalized medicine can significantly enhance the effectiveness of neuroprotective agents like citicoline, resveratrol, and *Ginkgo biloba* extract [37,87]. Patients with specific genetic profiles that influence oxidative stress pathways might derive more benefit from antioxidants such as resveratrol [130]. Additionally, genetic variations in the response to neurotrophic factors can guide the use of citicoline and other neuroprotective agents, ensuring that treatments are precisely tailored to support RGC survival [142].

Tailoring IOP-lowering therapies based on genetic insights also holds potential for enhancing treatment precision. Precision medicine can also refine the use of bioactive compounds that modulate IOP [143]. Genetic markers associated with aqueous humor dynamics can help predict which patients will respond best to bioactive compounds targeting the trabecular meshwork or ciliary body. For example, individuals with polymorphisms affecting the *CYP1B1* gene may exhibit differential responses to cannabinoids or melatonin in IOP regulation [144]. Tailoring IOP-lowering therapies based on genetic predispositions can enhance treatment efficacy and minimize adverse effects. Furthermore, personalized bioactive treatments targeting the inflammatory and oxidative stress components of glaucoma have shown promise in enhancing therapeutic efficacy. For instance, a study explored the role of neuroinflammation in glaucoma and identified specific molecular mechanisms involved in RGC death [145]. The researchers highlighted potential therapeutic targets, such as the adenosine A2A receptor and toll-like receptors (TLRs) 2 and 4, which are implicated in the inflammatory pathways of glaucoma. By developing treatments that specifically modulate these targets, it is possible to address the inflammatory and oxidative stress aspects of glaucoma more precisely, leading to improved patient outcomes. Additionally, patients with genetic variants in the Nrf2 pathway, a key regulator of antioxidant responses, may benefit more from bioactive compounds like sulforaphane, which activates this pathway [146]. Similarly, variations in genes related to inflammatory responses can guide the use of curcumin or resveratrol, optimizing their anti-inflammatory effects in specific patient populations.

These precision-guided strategies elevate the role of bioactive compounds from general adjuncts to targeted interventions. By integrating genetic profiling, clinicians can better predict which patients will benefit from specific compounds, reduce the risk of adverse effects, and monitor therapeutic response with greater accuracy. Ultimately, precision medicine enables a shift from trial-and-error prescribing toward rational, individualized glaucoma care, in which bioactive compounds are tailored to each patient’s molecular and clinical profile.

### 4.3. Biomarker Development and Monitoring

The success of precision medicine in bioactive glaucoma treatment hinges on the development and utilization of biomarkers [19]. These biomarkers provide real-time feedback on treatment efficacy, disease progression, and patient response, enabling dynamic adjustments to therapy. By offering insights into individual patient profiles, biomarkers can help optimize therapeutic strategies and improve clinical outcomes. Identifying molecular biomarkers associated with glaucoma can facilitate early detection and continuous monitoring [147]. For instance, the levels of specific proteins or microRNAs in ocular fluids can indicate disease activity and response to treatment [148]. Monitoring these biomarkers enables the tailoring of bioactive therapies to meet individual needs, ensuring that patients receive the most effective interventions.

Technological advancements in imaging further expand biomarker-based monitoring possibilities. Advancements in imaging technologies, such as optical coherence tomography (OCT) and confocal scanning laser ophthalmoscopy, have revolutionized the visualization of the optic nerve head and retinal layers [149,150]. Imaging biomarkers allow for the tracking of structural changes over time, providing valuable insights into the effectiveness of neuroprotective bioactive compounds. Personalized treatment regimens can be dynamically adjusted based on imaging outcomes, enhancing the precision and success of glaucoma management. Additionally, pharmacogenomic testing can identify genetic variants that influence drug metabolism and response [151]. Integrating pharmacogenomic data with bioactive treatments enables clinicians to predict how patients will metabolize and respond to specific compounds, reducing the risk of adverse effects and improving therapeutic outcomes by selecting bioactives most compatible with each patient’s genetic profile. For example, variations in the *CYP1B1* gene have been associated with different responses to certain glaucoma medications, highlighting the importance of considering genetic factors in treatment selection [152].

## 5. Challenges and future directions

### 5.1. Challenges with bioactive compounds

Despite the potential of bioactive treatments, several critical challenges must be addressed to enable their effective integration into glaucoma therapy. The multifactorial nature of glaucoma, characterized by complex interactions between genetic, environmental, and lifestyle factors, requires a deep understanding of these interactions and their implications for bioactive treatments.

Implementing precision medicine in glaucoma care also requires sophisticated data integration techniques, such as the integration of diverse data types, including genomic, proteomic, imaging, and clinical data. Advanced bioinformatics and machine learning techniques are essential for analyzing these vast datasets and identifying meaningful patterns that can guide personalized treatments. For example, the use of machine learning models, such as convolutional neural networks (CNNs), has been applied to OCT images to detect glaucomatous damage with high accuracy [18]. Translating these research findings into clinical practice involves overcoming numerous logistical, regulatory, and other challenges. Standardized protocols for genetic testing, biomarker analysis, and personalized treatment planning are crucial for the successful implementation of precision medicine in glaucoma care. The American Academy of Ophthalmology has published guidelines for genetic testing in eye disorders, emphasizing the importance of standardized approaches to ensure accurate and reliable results [153]. Additionally, patient engagement and education are vital components of this approach. Patients must be well-informed about the benefits and limitations of genetic testing and personalized treatments to ensure their participation and adherence to individualized treatment regimens. Educational resources provided by organizations like the Glaucoma Research Foundation offer valuable information to patients, helping them understand their condition and the available treatment options [154].

Therefore, precision medicine, combined with bioactive treatments, represents a transformative approach to glaucoma management. By tailoring therapies to individual genetic and phenotypic profiles, we can enhance the efficacy and safety of interventions. Ongoing research and technological advancements will continue to refine this approach, offering new hope for patients with glaucoma. As we move toward a more personalized healthcare paradigm, the integration of precision medicine with bioactive treatments holds the promise of significantly improving outcomes for those affected by this sight-threatening disease.

### 5.2. Comparative advantages of bioactive compounds over conventional therapies

Bioactive compounds may offer several advantages over conventional IOP-lowering medications [23]. First, many bioactives provide multimodal therapeutic effects—such as neuroprotection, antioxidant activity, mitochondrial support, and anti-inflammatory effects—that extend beyond IOP reduction, targeting the underlying pathophysiology of glaucomatous damage [22,86,88]. In contrast, conventional therapies primarily address aqueous humor dynamics and do not preserve RGCs. Second, some bioactive compounds (e.g., resveratrol, alpha-lipoic acid, citicoline) have shown favorable safety profiles and may be better tolerated over the long term [25,26,44,155]. Third, bioactives often target systemic or age-related pathways, which may be particularly beneficial in patients with comorbid neurodegenerative or vascular conditions [42,59,67]. Finally, the potential for personalization via precision medicine, including genetic-guided therapy selection, gives bioactives a unique role in future glaucoma care. However, these potential advantages must be weighed against limitations such as bioavailability and lack of regulatory standardization.

### 5.3. Ongoing clinical trials

As bioactive compounds move through clinical trials, evaluating their safety and efficacy in glaucoma management remains crucial. The path from preclinical promise to clinical application is fraught with challenges. This section examines ongoing clinical trials, discusses barriers to clinical translation, and explores the potential for combination therapies involving bioactive compounds in glaucoma treatment.

A number of clinical trials are exploring the therapeutic potential of bioactive compounds in glaucoma treatment. Several clinical trials are currently underway to evaluate the efficacy and safety of various bioactive compounds in glaucoma management [17,23,33,126]. A Phase II clinical trial (NCT02862938) is evaluating the efficacy of the NT-501 ECT implant in patients with glaucoma [156]. The NT-501 implant utilizes encapsulated human retinal pigment epithelial cells genetically engineered to secrete CNTF, a bioactive compound known for its neuroprotective properties. By delivering CNTF directly to the vitreous cavity of the eye, the implant aims to provide sustained neuroprotection to RGCs, potentially preserving visual function and optic nerve health in glaucoma patients [21]. Additionally, a phase II, randomized, double-blind, placebo-controlled trial (NCT02585540) is investigating the potential benefits of recombinant human nerve growth factor (rhNGF) in glaucoma patients, focusing on neuroprotective effects and visual field progression [21,157]. Similarly, a phase II/III trial (NCT01408472) is assessing the NT-501 CNTF implant in patients with POAG, aiming to evaluate improvements in RGC function and overall visual acuity [21]. Furthermore, a phase II trial (NCT00603485) is evaluating the brimonidine implant’s ability to lower IOP and preserve RGCs in patients with POAG [21,91]. Lastly, a phase IV, randomized trial (NCT00304729) is exploring the use of citicoline in glaucoma and ischemic optic neuropathy, with primary outcomes measuring visual function and retinal nerve fiber layer integrity [21,24,25]. These ongoing trials reflect the growing interest in bioactive compounds as potential adjunctive therapies in glaucoma management. The results of these studies will provide crucial insights into the clinical efficacy and safety of these compounds, potentially paving the way for their integration into standard glaucoma care.

In addition to the trials mentioned above, other studies are investigating bioactive compounds such as nicotinamide (vitamin B_3_) and Ginkgo biloba extract for their potential neuroprotective effects in glaucoma [158,159]. Nicotinamide is being evaluated in several pilot studies for its role in preserving retinal ganglion cell function through mitochondrial support [158]. Early findings suggest improvements in visual function metrics in glaucoma patients receiving high-dose nicotinamide supplementation. Similarly, Ginkgo biloba extract has shown promise in improving ocular blood flow and slowing visual field loss, particularly in normal-tension glaucoma. While many of these studies are in early phases or observational in nature, they contribute to a growing body of evidence supporting the integration of nutraceuticals and naturally derived bioactives in glaucoma care. Continued clinical validation through larger, controlled trials will be essential to confirm their safety, efficacy, and potential for regulatory approval.

### 5.4. Barriers to clinical translation

Despite the promising preclinical data and ongoing clinical trials, several barriers hinder the clinical translation of bioactive compounds in glaucoma therapy. Many bioactive compounds exhibit poor bioavailability when administered orally or have limited ability to penetrate ocular tissues in therapeutic concentrations [160]. Overcoming this barrier requires innovative drug delivery systems, such as nanoparticle formulations to enhance ocular penetration, sustained-release implants for long-term drug delivery, and novel topical formulations with improved corneal permeability [161,162].

Several cutting-edge ocular drug delivery platforms are being developed to address these limitations and improve the bioavailability of bioactive compounds. Nanoparticle-based systems, such as liposomes, polymeric nanoparticles, and solid lipid nanoparticles, enhance corneal penetration and prolong drug residence time on the ocular surface [161, 163]. For instance, curcumin-loaded nanoparticles have demonstrated improved stability and intraocular delivery in experimental models [164,165]. In situ gelling systems, which transition from liquid to gel upon contact with the eye, increase retention and reduce dosing frequency [166]. Microneedle arrays represent a minimally invasive strategy for delivering drugs directly into the suprachoroidal space or sclera, bypassing the corneal barrier [167]. Additionally, intravitreal implants such as sustained-release microspheres or nanofiber scaffolds are being explored to deliver compounds like resveratrol or citicoline over extended periods [168]. These innovations not only improve bioavailability but also enhance patient adherence and reduce systemic exposure, making them promising strategies for advancing bioactive compound-based glaucoma therapy.

Standardizing bioactive compounds, especially natural ones, also presents complex challenges in consistency and quality. Natural bioactive compounds, in particular, can vary significantly in composition and potency depending on their source and extraction methods [22,23]. Ensuring consistent therapeutic effects requires the development of standardized extraction and purification protocols, establishment of quality control measures for bioactive preparations, and creation of regulatory guidelines specific to bioactive-based therapies. Moreover, establishing the long-term safety and efficacy of bioactive compounds poses several challenges. Glaucoma is a chronic disease requiring lifelong management; the disease necessitates extended clinical trials to assess long-term outcomes, investigate cumulative side effects or drug interactions from prolonged use, and measure neuroprotective effects that shorter clinical trials cannot detect.

Navigating regulatory pathways and commercial viability further complicates clinical translation. The regulatory pathway for bioactive compounds, especially those derived from natural sources, can be complex and uncertain. Issues such as classification (e.g., drug vs. dietary supplement), varying regulatory requirements across different countries, and the need for extensive safety and efficacy data to gain regulatory approval present significant hurdles. Additionally, developing bioactive-based therapies for glaucoma can be costly, with uncertain commercial returns. High costs associated with large-scale, long-term clinical trials, challenges in patenting natural compounds or their derivatives, and potential market competition from generic drugs or established therapies are notable obstacles. Designing appropriate clinical trials to evaluate bioactive compounds in glaucoma presents unique challenges, including selecting relevant outcome measures for neuroprotection, determining appropriate trial duration to observe meaningful effects, and accounting for the multifactorial nature of glaucoma progression [86,130].

Taken together, these challenges explain the disconnect between promising results observed in preclinical glaucoma models and the limited clinical adoption of bioactive compounds. Translating these findings into viable therapies requires a concerted effort to improve formulation science, harmonize regulatory standards, and conduct long-term clinical studies in diverse patient populations. Without addressing these translational gaps, the therapeutic potential of bioactive compounds in glaucoma will remain largely unrealized.

### 5.5. Potential for combination therapies

The complex pathophysiology of glaucoma suggests that combination therapies, leveraging the diverse mechanisms of different bioactive compounds and conventional treatments, may offer superior outcomes [35]. Combining bioactive compounds with different neuroprotective mechanisms may provide enhanced RGC preservation. For example, citicoline supports neuronal membrane repair by increasing phosphatidylcholine synthesis, thereby stabilizing cell membranes and aiding in neuronal recovery [169]. When combined with resveratrol, known for its anti-apoptotic effects, this dual approach could synergistically improve RGC survival by simultaneously reinforcing cellular integrity and preventing programmed cell death [128]. The integration of coenzyme Q10 with citicoline offers a promising avenue by targeting mitochondrial health and enhancing neuroprotection, independent of IOP-lowering effects, potentially slowing the progression of glaucomatous neurodegeneration [170]. Such synergistic effects highlight the potential for innovative, multifaceted approaches in glaucoma management. Integrating bioactive compounds with conventional IOP-lowering medications may also offer more effective pressure control. A study found that Mirtogenol (a supplement similar to GBE) combined with latanoprost (a prostaglandin analog) had a synergistic effect, resulting in significantly lower IOP and improved retinal blood flow compared to latanoprost alone [171].

Combining bioactive compounds with different antioxidant and anti-inflammatory properties may provide broader protection against oxidative stress and inflammation [61,172]. For example, alpha-lipoic acid (direct antioxidant) combined with curcumin (NF-κB inhibition) may enhance the mitigation of oxidative damage and inflammatory processes [172]. Integrating bioactive compounds with existing glaucoma medications may enhance overall treatment efficacy. Another study showed omega-3 fatty acids (neuroprotection) combined with brimonidine may improve IOP control and enhance neuroprotection [21]. Tailoring combination therapies based on individual patient characteristics and glaucoma subtypes may optimize treatment outcomes, such as customized combinations for normal-tension glaucoma versus high-tension glaucoma, potentially resulting in more targeted and effective management strategies.

These examples demonstrate that bioactive compounds, when used in combination with other neuroprotective agents or conventional IOP-lowering therapies, can yield synergistic effects on RGC survival, IOP control, and vascular regulation. The rationale for such combinations lies in their ability to simultaneously target multiple pathogenic pathways—including oxidative stress, mitochondrial dysfunction, inflammation, and excitotoxicity. Several combinations, such as citicoline with coenzyme Q10, or resveratrol with curcumin, have shown promise in preclinical models and small clinical trials [61]. Going forward, optimizing these therapeutic pairs based on patient subtype and genetic profiles will be central to realizing their full potential in clinical glaucoma care.

## 6. Conclusion

The future of bioactive compounds in glaucoma therapy holds immense promise, particularly when integrated with precision medicine strategies that tailor treatments to individual genetic and phenotypic profiles. These compounds offer the potential to transform glaucoma management by complementing IOP-lowering therapies with neuroprotective, antioxidative, and anti-inflammatory mechanisms. Ongoing clinical trials are beginning to generate valuable data on their therapeutic effects.

However, realizing this potential requires overcoming several critical barriers to clinical translation, including limited bioavailability, variability in compound standardization, long-term safety concerns, and complex regulatory pathways. The development of innovative drug delivery platforms and robust clinical trial designs will be key to addressing these challenges. Furthermore, combination therapies that strategically pair bioactive compounds with conventional or targeted treatments offer exciting prospects for more comprehensive and personalized disease management.

Moving forward, collaborative efforts between academic researchers, clinicians, pharmaceutical developers, and regulatory agencies will be essential to ensure that emerging therapies are both scientifically rigorous and clinically viable. With continued research, cross-disciplinary innovation, and adequate funding, bioactive-based treatments may soon become valuable additions to the glaucoma treatment arsenal—ultimately improving patient outcomes and addressing the unmet needs in neurodegenerative ocular care.

## Figures and Tables

**Table 1:** Natural Bioactives for Glaucoma Treatment.

Bioactive Compound	Source	Mechanism of Action	Potential Benefits	Reported Dosage	Ref.

***Ginkgo biloba* extract**	Leaves of Ginkgo biloba	Antioxidant, vasodilatory effects	Protects RGCs from oxidative stress, enhances blood flow	120 mg/day (40 mg three times daily)	[27,36,37]
**Resveratrol**	Grapes, berries, peanuts	Antioxidant, anti-inflammatory, improves mitochondrial function	Protects RGCs, reduces IOP, improves ocular blood flow	250–500 mg/day	[26,41,45]
**Curcumin**	Turmeric (Curcuma longa)	Anti-inflammatory, antioxidant, neuroprotective	Reduces inflammation, protects RGCs, improves mitochondrial function	3.6 g/day	[46,47,49]
**Cannabidiol (CBD)**	Cannabis	Anti-inflammatory, antioxidant, neuroprotective	Reduces IOP, protects RGCs, improves ocular blood flow	20–40 mg sublingual; note: 40 mg may increase intraocular pressure	[55–57]
**Alpha-lipoic acid (ALA)**	Synthesized in mitochondria	Antioxidant, supports cellular energy production	Protects RGCs, improves visual function, reduces oxidative stress	150–600 mg/day	[28,61,63]
**Bilberry Extract**	Bilberry fruit	Contains anthocyanins with antioxidant properties	Protects RGCs from oxidative stress; may improve ocular blood flow and reduce IOP	60 mg twice daily	[68,69]
**Astaxanthin**	Microalgae, yeast, salmon	Powerful antioxidant, anti-inflammatory effects	Protects RGCs from oxidative stress and inflammation; potential neuroprotective effects	2–12 mg/day	[70,71]
**Green Tea Polyphenols (EGCG)**	Green tea leaves (Camellia sinensis)	Strong antioxidant and anti-inflammatory effects	Reduces IOP; protects RGCs and enhances ocular blood flow	400 mg/day	[23,72]
**Ginseng**	Root of the ginseng plant	Contains ginsenosides with antioxidant, anti-inflammatory, and neuroprotective properties	Enhances retinal function, protects against oxidative damage, may reduce IOP	200–400 mg/day	[75–77]
**Saffron**	Stigma of the saffron crocus flower	Rich in crocin, modulates inflammatory pathways	Protects RGCs from apoptosis; may enhance retinal health and function	30 mg/day	[78,79]
**Quercetin**	Onions, apples, berries	Flavonoid with strong antioxidant, anti-inflammatory, and neuroprotective properties	Protects RGCs from oxidative damage and apoptosis; may reduce IOP and inflammation	30–50 mg/day	[73,74]
**Omega–3 Fatty Acids**	Fish oil, flaxseed oil, chia seeds	Anti-inflammatory and neuroprotective effects	May reduce IOP, protect RGCs, and improve retinal function	1000–2000 mg/day	[80,81]

**Table 2:** Synthetic Bioactives for Glaucoma Treatment.

Bioactive Compound	Class	Mechanism of Action	Potential Benefits	Reported Dosage	Ref.

**Memantine**	NMDA Receptor Antagonist	Blocks excessive calcium influx, prevents excitotoxicity	Protects RGCs from death, potential neuroprotection	20 mg/day	[88,89]
**Brimonidine**	Alpha–2 Adrenergic Agonist	Upregulates neurotrophic factors, reduces oxidative stress	Preserves visual field function, reduces IOP	0.2 % eye drops, twice daily	[90,91]
**Netarsudil**	Rho Kinase (ROCK) Inhibitor	Promotes RGC survival, enhances axon regeneration	Improves ocular blood flow, reduces IOP	0.02 % ophthalmic solution, once daily	[33,126,127]
**Citicoline**	Neuroprotective Agent	Enhances mitochondrial function, reduces oxidative stress	Improves visual function, supports RGC survival	500–1000 mg/day oral; 2 % eye drops	[24,25,128]
**MitoQ**	Mitochondria-targeted Antioxidant	Accumulates in mitochondria, protects against oxidative stress	Protects RGCs from oxidative damage	10 mg/day	[104]
**Bardoxolone methyl (RTA 408)**	Nrf2 Activator	Activates cellular antioxidant defenses	Protects against oxidative stress-induced RGC damage	10–20 mg/day	[105]
**XPro1595**	TNF-α Inhibitor	Reduces neuroinflammation	Protects RGCs, reduces RGC loss	1 mg/kg, subcutaneous injection (preclinical studies)	[111]
**Anakinra**	IL–1 Receptor Antagonist	Modulates neuroinflammation	Reduces neuroinflammation, potential neuroprotection	100 mg/day, subcutaneous injection	[112]
**Brinzolamide**	Carbonic Anhydrase Inhibitor	Inhibits carbonic anhydrase, reducing aqueous humor production	Lowers IOP effectively	1 % eye drops, twice daily	[113,114]
**Latanoprostene bound**	Prostaglandin Analog/Nitric Oxide Donor	Dual mechanism: increases uveoscleral and trabecular outflow	Significantly reduces IOP	0.024 % eye drops, once daily	[115,116]
**Sunitinib**	Tyrosine Kinase Inhibitor	Inhibits tyrosine kinases, reducing glaucomatous damage	Provides neuroprotection, may slow progression	50 mg/day (for oncology); ocular dosage not established	[117]
**Dorzolamide**	Carbonic Anhydrase Inhibitor	Reduces aqueous humor production	Effectively lowers IOP	2 % eye drops, twice daily	[118–120]
**Anecortave acetate**	Angiostatic Corticosteroid Analog	Inhibits angiogenesis and fibroblast activity	Lowers IOP	15 mg posterior juxtascleral injection every 6 months (investigational)	[121]
**Tafluprost**	Prostaglandin Analog	Increases uveoscleral outflow	Lowers IOP with fewer side effects	0.0015 % eye drops, once daily	[122,123]
**ILUVIEN^®^**	Corticosteroid Implant	Provides sustained release of corticosteroid	Controls inflammation and IOP over a long duration	0.19 mg intravitreal implant, releases 0.2 μg/day over 36 months	[124]

## Data Availability

The data that support the findings of this study are available from the corresponding author, Joan M. O’Brien, upon reasonable request.
